# Sleep, evening light exposure and perceived stress in healthy nulliparous women in the third trimester of pregnancy

**DOI:** 10.1371/journal.pone.0252285

**Published:** 2021-06-03

**Authors:** Randi Liset, Janne Grønli, Roger E. Henriksen, Tone E. G. Henriksen, Roy M. Nilsen, Ståle Pallesen

**Affiliations:** 1 Department of Psychosocial Science, Faculty of Psychology, University of Bergen, Bergen, Norway; 2 Department of Biological and Medical Psychology, Faculty of Psychology, University of Bergen, Bergen, Norway; 3 Faculty of Health and Social Sciences, Western Norway University of Applied Sciences, Bergen, Norway; 4 Division of Mental Health Care, Valen Hospital, Fonna Local Health Authority, Valen, Norway; 5 Department of Clinical Medicine, Section for Psychiatry, Faculty of Medicine, University of Bergen, Bergen, Norway; 6 Norwegian Competence Center for Sleep Disorders, Haukeland University Hospital, Bergen, Norway; 7 Optentia, The Vaal Triangle Campus of the North-West University, Vanderbijlpark, South-Africa; IRCCS Istituto Delle Scienze Neurologiche di Bologna, ITALY

## Abstract

**Objective:**

Sleep disturbances are common in pregnancy, and the prevalence increases during the third trimester. The aim of the present study was to assess sleep patterns, sleep behavior and prevalence of insomnia in pregnant women in the third trimester, by comparing them to a group of non-pregnant women. Further, how perceived stress and evening light exposure were linked to sleep characteristics among the pregnant women were examined.

**Methods:**

A total of 61 healthy nulliparous pregnant women in beginning of the third trimester (recruited from 2017 to 2019), and 69 non-pregnant women (recruited in 2018) were included. Sleep was monitored by actigraphy, sleep diaries and the Bergen Insomnia Scale. The stress scales used were the Relationship Satisfaction Scale, the Perceived Stress Scale and the Pre-Sleep Arousal Scale. Total white light exposure three hours prior to bedtime were also assessed.

**Results:**

The prevalence of insomnia among the pregnant women was 38%, with a mean score on the Bergen Insomnia Scale of 11.2 (SD = 7.5). The corresponding figures in the comparing group was 51% and 12.3 (SD = 7.7). The pregnant women reported lower sleep efficiency (mean difference 3.8; 95% CI = 0.3, 7.3), longer total sleep time derived from actigraphy (mean difference 59.0 minutes; 95% CI = 23.8, 94.2) and higher exposure to evening light (mean difference 0.7; 95% CI = 0.3, 1.2), compared to the non-pregnant group. The evening light exposure was inversely associated with total sleep time derived from actigraphy (B = -8.1; 95% CI = -14.7, -1.5), and an earlier midpoint of sleep (B = -10.3, 95% CI = -14.7, -5.9). Perceived stressors were unrelated to self-reported and actigraphy assessed sleep.

**Conclusion:**

In healthy pregnant participants sleep in the third trimester was preserved quite well. Even so, the data suggest that evening light exposure was related to shorter sleep duration among pregnant women.

## Introduction

Pregnancy represents a time of several significant changes in terms of physical, hormonal, and psychological alterations, all which may influence sleep. Accordingly, disturbed sleep is frequent during pregnancy [1]. Some sleep characteristics seem to be more frequently occurring in specific phases of the pregnancy; such as increased demand for sleep in the first trimester, and disrupted sleep in the third trimester. By the start of gestational week 40 a high percentage (75–98%) report multiple nocturnal awakening [2–4] Further, sleep quality has been found to worsen with age of the pregnant women [5]. Overall, there seems to be three main groups of sleep disturbances which predominate during pregnancy; 1) obstructive sleep apnea (OSA), 2) restless legs syndrome (RLS) and 3) insomnia. The latter is characterized by difficulty initiating sleep, nocturnal awakenings, or early-morning awakenings, occurring for at least three nights per week [1].

Insomnia has been reported by 62% of pregnant women, a number that is significantly higher than found in the general population (10–15%) [6]. Disrupted sleep among pregnant women may be caused by several hormonal and mechanical influences, and factors such as nocturia, dyspnea, nasal congestion, muscular aches and pelvic pains, fetal activity, leg cramps as well as gastric reflux [1]. There is evidence suggesting that poor sleep quality and insomnia negatively could influence the health of mother and offspring, e.g. in terms of preeclampsia [1], gestational diabetes mellitus, preterm birth [7], prolonged labor, increased pain during labor, Caesarean section, low birth weight [1, 8], depressive reactions [6, 8], and postnatal depression [9]. Thus, assessment of sleep and identifying predictors of poor sleep in pregnancy are clearly warranted.

Sleep is closely linked to circadian rhythms [10]. The central pacemaker, the suprachiasmatic nucleus (SCN) situated in the hypothalamus, is the master clock, and influences not only the sleep-wake rhythm, but also a vast array of physiological (e.g. hormonal) and behavioral (e.g. feeding) rhythms [11]. Light is the strongest “zeitgeber” and entrains the SCN to the 24-hour dark-light cycle. Information about environmental illumination is signaled to the SCN by melanopsin-containing intrinsically photoresponsive retinal ganglion cells (ipRGC) [12]. The SCN projects further to the pineal gland, producing the sleep promoting night hormone melatonin. Exposure to nocturnal light has been found to suppress melatonin secretion, a signal of darkness and marker for circadian rhythms [13]. A recent review reported that circadian rhythm disturbances, in both mother and infant in the postpartum period, are strongly correlated with maternal exposure to light [14]. Preclinically observations find that constant light exposure in pregnant rats reduced melatonin level and affected the pregnant progress negatively [15]. Especially light exposure in the evening and during the night increases alertness, disturbs sleep and delays the circadian rhythm [16]. In line with this, a review showed that avoidance of evening light was associated with less sleep disturbance and increased total sleep time in a non-pregnant population [17].

There is so far dearth of knowledge about how light exposure in pregnancy influence sleep and the circadian rhythm. A study of primipara pregnant women exposed for ocular blue/green light for two hours prior to and after bedtime (total of four hours) showed lower melatonin concentration than the control group (red light) [18]. Natural light exposure at night showed an inversely association with sleep duration in pregnant women, assessed at first and third trimester [19]. Further, in agreement with this, a study of nocturnal artificial outdoor light (skyglow, light pollution) found a negative association with sleep duration in pregnant women [20].

Pregnancy is regarded as a vulnerable phase in life. Several aspects of pregnancy may increase perceived stress. One study showed that 78% of pregnant women experienced low to moderate levels and 6% reported high levels of psychosocial stress [21]. In agreement with this, a cohort study reported that women perceived more stress during pregnancy than during the postpartum period [22]. Stress affects sleep, and women with stress-related sleep disturbances during pregnancy are more likely to experience insomnia [23] and psychiatric disorders, compared to women without stress related sleep disturbance [23, 24]. Maternal prenatal stress can affect the physiological [7] and psychological health [25] of the pregnant women, and has been associated with negative fetal outcome such as stillbirth, preterm birth, intrauterine growth restriction and developmental delay [26–28], neurodevelopment [7, 29] as well as infectious disease in the offspring [30]. Pregnancy typically include worries about the health of the fetus, diet, weight gain, appearance, labor and delivery [27]. It can also be influenced by many other factors, including life events, social support, income level, educational background and partner relationship quality [31]. Marital distress may activate the central stress response system, the hypothalamic-pituitary-adrenal (HPA) axis and predict adverse stress effects. Satisfaction with partner on the other hand, may reduce stress activation [32].

Although some studies have attested to worsening of sleep in the third trimester, most of these studies are based on questionnaires. Few studies have combined sleep diaries with objective measures, such as actigraphy, when assessing sleep during pregnancy. Further, there is a dearth of knowledge when it comes to how light exposure in pregnancy is related to sleep disturbances. In addition, few studies have assessed how sleep in pregnant women are affected by different perceived stressors.

Against this background, the aim of the present study was to describe sleep patterns and sleep related behavior as well as the prevalence of sleep disturbances in population of pregnant women in the third trimester. Secondly, we aimed to compare them to a contrast group of non-pregnant women. Thirdly, we aimed to investigate how evening light exposure and perceived stress were associated with sleep characteristics among the pregnant women.

## Method

### Study population and design

The current study was observational, based on data collected as part of a randomized clinical trial, registered at ClinicalTrials.gov (NCT03114072), comparing sleep and light exposure data in pregnant women in the third trimester with female non-pregnant students.

**Fig 1:**
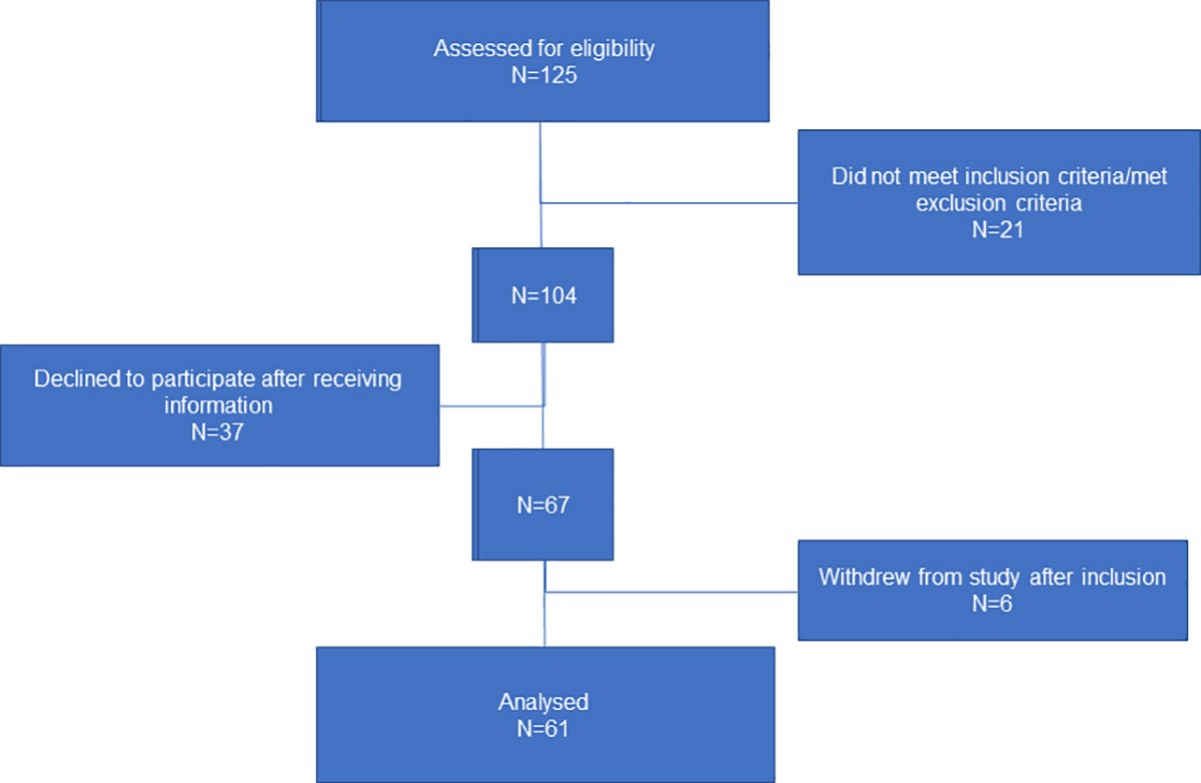
Flowchart of enrollment of pregnant women in the study.

Fig 1 presents a flowchart of enrollment of the pregnant women to the present study. Between May 2017 and April 2019 healthy nulliparous women were recruited during their standard health control about gestational week 24 by consulting midwives at antenatal-healthcare centers in the Municipality of Bergen, Norway. The midwives provided oral and written information about the study. If the pregnant women consented to receive more information or participate, further information was provided by the research team. Inclusion criteria were: 1) Nulliparous women, 2) expecting one child, 3) being in the third trimester of a normal pregnancy, 4) able to wear an actigraph during daytime and nighttime for the study period (one week) and, 5) able to complete a questionnaire in Norwegian. Exclusion criteria were; 1) somatic or psychiatric disorders, 2) fever and other health conditions affecting sleep, 3) working at night during the study protocol or 4) having an eye-condition affecting the translucency of the eyes. A total of 125 pregnant women were assessed for eligibility. After excluding those who declined participation, a final sample of 61 pregnant women were retained (Fig 1). The pregnant women finally started their participation with data collection between pregnancy week 28 to 32, mean week 29+0.

To compare sleep data and light exposure data with a non-pregnant population, we used data from a group of 69 healthy young women who were students at University of Bergen, during the period of February/March, August/September and October/November in 2018. Inclusion criteria were: 1) healthy women, 2) not pregnant, 3) able to wear an actigraph during daytime and nighttime for the study period (about one week) and, 5) able to complete questionnaires in Norwegian. We excluded those reporting sicknesses during the study-period.

### Variables

#### Demographic

Self-report questions were used to obtain information about maternal age, marital/partner status (married, cohabitating, single, separated/divorced, widow), level of education (high school and below, college and above), income (NOK<150 000–399 999, 400 000–599 999, 600 000–799 999, 800 000–1 mill and above; 10 NOK ≈ 1 US $), number of people living together in the household (partner, parents, parents in law, children, none, other) and smoking (daily, less than daily, never).

#### Subjective measure of sleep

*A sleep diary* was completed every morning. The sleep diary included items on number and duration of naps during the day, use of sleep medication (yes/no), bedtime (hh.mm), lights-out time (hh.mm), sleep latency (min), number of nocturnal awakenings, wake after onset sleep (WASO), waking and rise time (hh.mm). Included were also items assessing sleep quality and daytime sleepiness [33]. The outcomes used in the present study were total sleep time (TST), sleep efficiency (SE%) (total sleep time/ time in bed * 100) and midpoint of sleep (TST:2).

*The Bergen Insomnia Scale (BIS)* was administered at study initiation. Originally the time frame was last month, but this was changed to last week in order to capture the rapid change in sleep that may occur during pregnancy and also to align the BIS with the time frame for the sleep diary and actigraphy measures. The BIS consists of six items. The first four pertain to sleep onset, maintenance, early morning wakening insomnia, and not feeling restored after sleep. The last two assess level of daytime impairment due to poor sleep and dissatisfaction with sleep. Each item is rated on a scale ranging from 0 to 7 days per week, providing a composite score ranging from 0 to 42. An insomnia diagnosis was made if the participant scored at least 3 points on at least one of the first four items of the BIS, and scored at least 3 points on at least one of the last two items of the BIS [34]. Cronbachs alpha for the BIS was .80 for pregnant and .82 for non-pregnant women.

#### Objective measure of sleep and light

*Actigraphy*. To objectively estimate sleep patterns and light, each participant was asked to wear a commercially available wrist actigraph (Actiwatch Spectrum; Philips Respironics Inc.), on their non-dominant wrist, continuously throughout the study period. The actigraph registered movements by a piezoelectric accelerometer and epoch length was set to thirty seconds and the sensitivity was set to medium. An embedded light sensor in the actigraph recorded and determined the light exposure, expressed in illuminance (lux). The participants were instructed to press the event button on the actigraph to indicate when they turned off the light and tried to sleep, and when they finally woke up in the morning. Data was converted to objective sleep parameters through the Actiware (version 6.0.9, Philips Respironics Inc.) software. Rest intervals were manually scored based on raw data, reflecting motor activity, light exposure, event button presses and also supported by sleep diary data according to description of the criteria for defining the duration of the sleep episode [35]. Duration of the sleep episode is the sum of sleep onset latency (SOL) + total sleep time (TST) + time awake after sleep onset but before final awakening (WASO) + time in bed after the final awakening [36]. In cases where event button markers were not pressed or discrepancies between the sleep diary data and actigraph data were evident, duration of the sleep episode was set based on motor- activity. Three sleep related outcome variables were derived: Total sleep time (TST), sleep efficiency (SE) and midpoint of sleep. Midpoint of sleep provides an indirect measure of circadian phase [37]. Data on the total white light exposure during the three last hours before bedtime, were also retained and analyzed. Total white light refers to the total light illuminance (lux/m2). In the Actiwatch Spectrum device this measure is calculated from the integration of data from three color diode sensors detecting ambient light in the range from 400 nm to 700 nm [38].

#### Measure of stress

*Pre-Sleep Arousal Scale (PSAS)* was completed every night before bedtime. The PSAS assesses the state of psychophysiological arousal before sleep. The scale consists of 16 items and measures somatic (e.g. heart racing, shortness of breath, stomach upset) as well as cognitive (e.g. worry about falling asleep, depressing or anxious thoughts, mentally alert) components of arousal. Responses are recorded on a five-point Likert scale ranging from 1 (not at all) to 5 (extremely), providing a composite score ranging from 16 to 80. Higher scores indicate higher states of arousal [39]. Cronbachs alpha for the PSAS scale was on average for all the seven days .76.

*Perceived Stress Scale (PSS)* was measured at study initiation. The PSS measures how often a situation in one’s life has been considered stressful during the previous week and consists of 10 items (e.g. “…upset of unexpected happening” and “…nervous and stressed”). Each item is scored on a five-point Likert-scale ranging from 1 (never) to 5 (very often), with a total score ranging from 10 to 50. Four items are reversed, and higher scores indicate higher levels of stress [40]. Cronbachs alpha for the PSS was .64 in the present study.

*Relationship Satisfaction Scale (RSS)* was also administered at the start of the data collection. This instrument measures marital satisfaction and relationship quality. It consists of 10-items (e.g. “Have a close relationship with my spouse/partner” and “My partner and I have problems in our relationship”). One response option was by inadvertence lost in the process of finalizing the questionnaire, hence, a 5-point scale ranging from 1 (strongly agree) to 5 (strongly disagree), providing a composite score ranging from 10 to 50 where used rather a 6-point scale. Three items were reversed, and higher scores represent lower levels of relationship satisfaction [41]. Cronbachs alpha for the RSS scale was .74.

### Statistical methods

All analyses were performed using IBM SPSS Statistics version 25 (SPSS, Chicago, IL, USA), and Stata IC version 16 (Stata Statistical Software, College Station, TX, USA) and R version 3.5.1 [42]. Notably, data on TST, SE, and midpoint of sleep were collected both from sleep diary and actigraphy, whereas data on white light were exclusively collected from actigraphy. In terms of actigraphy there were valid data for 54 pregnant and 58 non-pregnant women, yielding a somewhat lower sample size compared with the sample with valid sleep diary data. Descriptive statistics were used to summarize demographic characteristics of the sample, presented as means and percentages, and differences in age, alcohol and BIS-scores between pregnant and non-pregnant women were examined by independent samples t-tests and presented as means (SDs) and p-values.

Linear mixed effects models were used for comparing the outcomes TST, SE, midpoint of sleep, and total white light between pregnant and non-pregnant women. All models included pregnancy group, weekday, and a group-by-weekday interaction as independent categorical terms. To account for repeated measures and the intra-individual correlation across weekdays, we specified a random intercept for the individual using the individual’s unique study number. For some outcomes, we also added a first-order autoregressive error term to account for lagged correlation not captured by the random intercept alone. The group difference for each outcome was estimated for each weekday as well as across all weekdays overall. For the overall analysis, the group-by-weekday interaction was omitted from the models. To examine potential group-by-weekday interactions, we further used likelihood ratio tests. All analyses were performed crude and with adjustment for age, weekday and calendar month.

We also used linear mixed effects models to examine if the exposures RSS, PSS, PSAS, and total white light were associated with the outcomes TST, SE and midpoint of sleep measures, both with sleep diaries and actigraphy. These analyses were carried out only for data on the pregnant women. The analysis of each exposure on each outcome was performed crude and with adjustment for age, weekday and calendar month and a random intercept for the individual was specified to account for the repeated measures and intra-individual correlation across weekdays. Note, that in all abovementioned analyses, we excluded outliers in both outcomes and exposures. In addition, total white light was strongly right-skewed and was therefore log-transformed before entering the models. Furthermore, consistency for total scores on RSS, PSS, and PSAS was estimated by Cronbach’s alpha.

### Ethical considerations

The study was approved by the Regional Committee for Medical and Health Related Ethics, in Western Norway (2016/1394/REK vest). All participants provided written informed consent before inclusion in the study.

## Results

In the 61 healthy primipara pregnant women, the mean age was 30.6 years (SD 4.0, range 24–43), whereas the mean age of the 69 healthy non-pregnant group age was 23.1 years (SD 2.8, range 19–33), p < .001.

Sample characteristics in terms of marital status, education, economics (total income in the household), number of adults and children living in the household for the pregnant women are displayed in Table 1. In all, 96.7% of the primipara women were married or living with a partner, 84% had education at least at college level, 72% had an income of 800 000 NOK (≈ 80 000 US $) or more, and 95% were living with their partner. Only one pregnant woman reported she was smoking. None of the pregnant women reported consumption of alcohol during the study week, compared to 9 of the non-pregnant group.

**Table 1:** Demographic factors among pregnant and non-pregnant women, self-reported data.

Factor	Level	Pregnant	Non-pregnant	P-value
N		61	69	
Age, mean (SD)		30.6 (4.0)	23.1 (2.8)	< .001
Marital status, n (%)	Married	18 (30%)		
	Cohabitating/partner	41 (67%)		
	Single	2 (3%)		
Education, n (%)	< = Senior high school	10 (16%)		
	College and above	51 (84%)		
Economics, n (%)	<150 000–399 999 NOK	6 (10%)		
	400 000–599 999 NOK	4 (7%)		
	600 000–799 999 NOK	7 (11%)		
	800 000–1mill. NOK or above	44 (72%)		
Adult, total in Household, n (%)	1	2 (3%)		
	2	58 (95%)		
	4	1 (2%)		
Children, total in household, n (%)	0	59 (97%)		
	1	1 (2%)		
	3	1 (2%)		
Smoking, n (%)	Daily	1 (2%)		
	Not at all	60 (98%)		
Alcohol, n (%)	Units 1–6	0 (0)	9 (6.2)	< .001
Physical activity, minutes, mean (SD)		20.9 (27.5)		
Relaxing activity, minutes, mean (SD)		4.5 (13.3)		
Bergen Insomnia Scale, mean (SD)		11.2 (7.5)	12.3 (7.7)	.943^b^
Insomnia, (%)		(38%)	(51%)	.136

N = number of participants; SD = standard deviation; NOK = Norwegian kroner; 10 NOK ≈ 1 US $.

^a^N = 67

^b^Adjusted for age.

The pregnant group had a mean score of 11.2 (SD 7.5) on the BIS, whereas the mean BIS score for the non-pregnant group was 12.3 (SD 7.7) which did not amount to a significant difference neither before nor after adjusting for age. In the pregnant group, 38% scored above cut-off for insomnia compared to 51% in the non-pregnant group.

Table 2 displays the results from linear mixed models regarding sleep and evening light exposure variables for each weekday. The sleep diary data showed no significant difference between the non-pregnant women and the pregnant women in terms of TST or midpoint of sleep after adjustment for age, weekday and calendar month. However, the pregnant group had lower SE compared to the non-pregnant group, both in the crude, mean difference = 3.4 (95% CI = 1.3, 5.6), and in the adjusted analysis, mean difference = 3.8 (95% CI = 0.3, 7.3).

**Table 2:** Sleep and light exposure variables for each weekday, and the association between pregnant and non-pregnant women.

		Pregnant		Non pregnant	Crude model:	Adjusted model:
	N	Mean (SE)	N	Mean (SE)	Mean difference (95% CI) [P-value]	Mean difference (95% CI) [P-value]^a^
**Total sleep time** (Self-report)						
Average across week	61	444.2 (5.9)	52	461.0 (5.8)	16.6 (0.3, 32.9) [.046]	-14.1 (-40.8, 12.6) [.301]
**Sleep efficiency** (Self-report)						
Average across week	61	86.2 (0.7)	52	89.6 (0.8)	3.4 (1.3, 5.6) [.002]	3.8 (0.3, 7.3) [.033]
**Midpoint of sleep** (Self-report)						
Average across week	61	03:52 (00:05)	52	04:40 (00:08)	00:47 (00:27, 01:08) [< .001]	00:21 (-00:14, 00:56) [.225]
**Total sleep time** (Actigraph)						
Average across week	55	463.0 (5.5)	58	448.4 (7.0)	-14.7 (-32.3, 2.9) [.102]	-59.0 (-94.2, -23.8) [< .001]
**Sleep efficiency** (Actigraph)						
Average across week	55	85.9 (0.7)	58	85.0 (0.7)	-1.0 (-3.0, 1.1) [.357]	-0.3 (-4.3, 3.6) [.862]
**Midpoint of sleep** (Actigraph)						
Average across week	55	04:24 (00:03)	58	05:01 (00:06)	00:36 (00:21, 00:51) [< .001]	-00:04 (-00:33, 00:26) [.831]
**Total white light log** transformed						
Average across week	54	8.4 (0.1)	58	7.0 (0.1)	-1.4 (-1.6, -1.1) [< .001]	-0.7 (-1.2, -0.3) [.002]

Estimated by linear mixed effects models without the group-by-weekday interaction were used for comparing the outcomes TST, SE, Midpoint of sleep, Total White light between pregnant and non-pregnant women. N = number of participants; SE = standard error; CI = confidence interval.

^a^ Adjusted for age, weekday and calendar month.

In terms of the actigraph data, the non-pregnant women had 59 minutes shorter TST than the pregnant women in the adjusted analysis (Table 2). No group differences were found for SE or midpoint of sleep after adjustment for age, weekday and calendar month. Compared to the non-pregnant women, the pregnant women were exposed to more total white light, the three last hours before bedtime, also after adjustment for age, weekday and calendar month.

**Fig 2:**
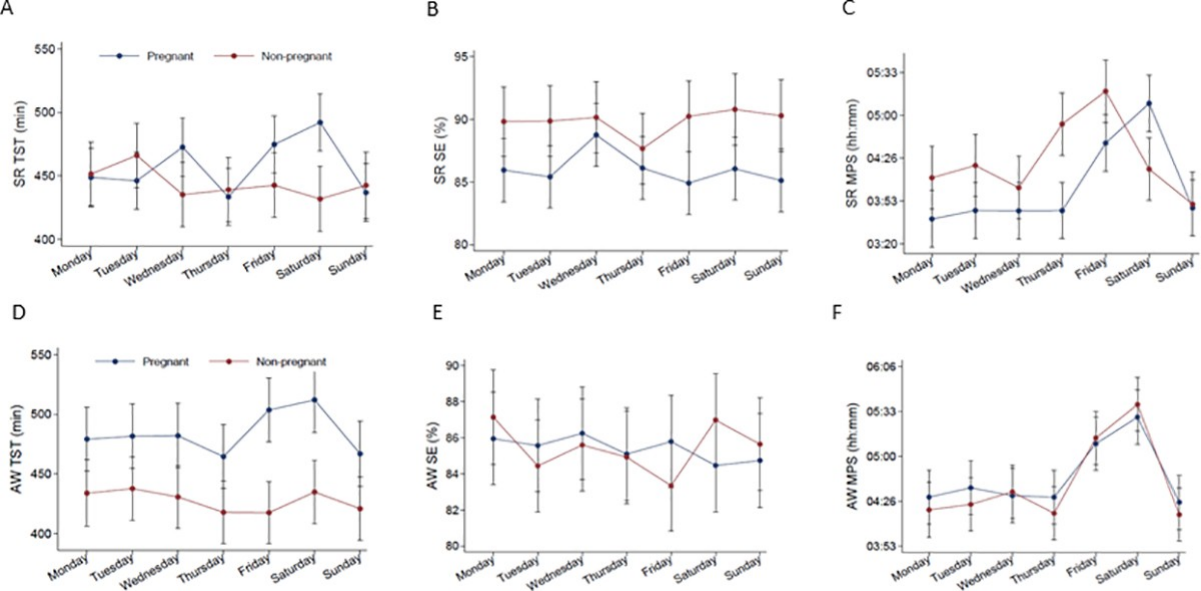
Daily sleep, in pregnant and non-pregnant women. Estimated by linear mixed effects models including group-by-weekday interaction. The p value for time-by-group interaction was (A) p < .001 for Self-reported (SR) total sleep time (TST), (B) p = .192 for Self-reported sleep efficiency (SE), (C) p < .001 for Self-reported midpoint of sleep (MPS) was, (D) p = .183 derived from actigraphy (AW) for total sleep time (TST), (E) p = .027 derived from actigraphy for sleep efficiency (SE), (F) p = .408 derived from actigraphy for midpoint of sleep.

**Fig 3:**
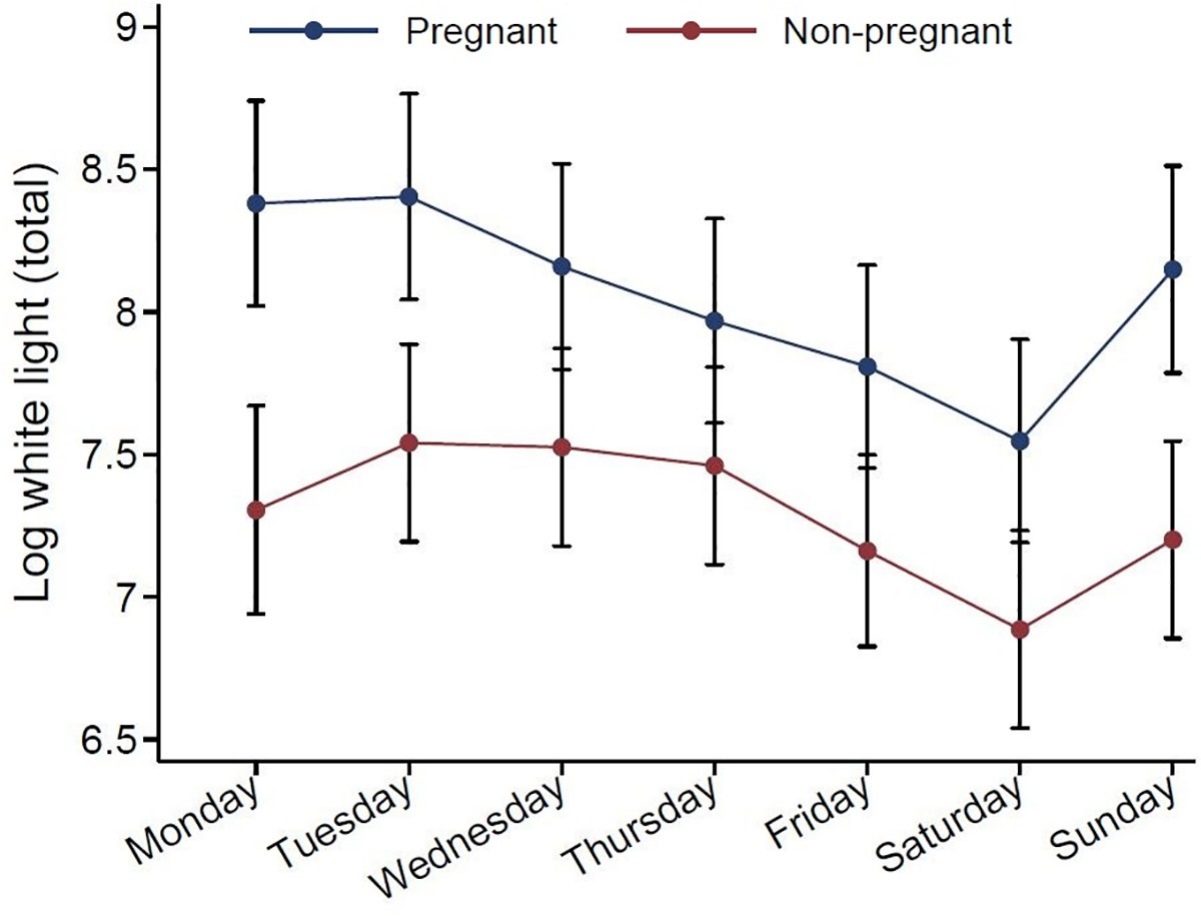
Daily evening light exposure, in pregnant and non-pregnant women. Estimated by linear mixed effects models including group-by-weekday interaction. The p value for time-by-group interaction was p = .307 derived from actigraphy for total white light (log transformed).

Daily change in sleep, and evening light exposure in pregnant and non-pregnant women, are displayed in Figs 2 and 3, analyzed by linear mixed models, and adjusted for age and calendar month. Self-reported data TST and midpoint of sleep showed a different pattern over the days for the two groups (p for interaction < .001), but not in SE. For actigraph data, SE showed a different between the groups (p for interaction .027), but daily change in TST, midpoint of sleep and total white light showed a similar pattern in the two groups.

Table 3 displays the association between the exposures RSS, PSS, PSAS and the outcomes TST, SE and midpoint of sleep, in pregnant women, analyzed by linear mixed model. When using self-reported data, an association between PSAS and midpoint of sleep was only detected before, but not after adjustment for age, weekday and calendar month. The exposures RSS, PSS, PSAS did not show any relationship with any of the sleep variables TST, SE and midpoint of sleep, neither on sleep diary nor actigraphy data before or after adjustment.

**Table 3:** Association between sleep and different types of perceived stressors in pregnant women.

	Self-reported				Actigraphy			
	Crude model:		Adjusted model:^a^		Crude model:		Adjusted analysis:^a^	
	Beta	95% CI [P]	Beta	95% CI [P]	Beta	95% CI [P]	Beta	95% CI [P]
**Total sleep time**								
RSS	-3.3	-8.1, 1.5 [.178]	-2.7	-7.4, 2.0 [.265]	-2.8	-7.4, 1.7 [.226]	-2.6	-7.0, 1.7 [.238]
PSS	-0.2	-2.8, 2.4 [.868]	-0.6	-3.0, 1.9 [.646]	0.3	-2.1, 2.7 [.785]	1.2	-1.0, 3.5 [.270]
PSAS	-2.0	-4.3, 0.2 [.078]	-1.9	-4.1, 0.2 [.075]	-0.7	-2.7, 1.3 [.468]	-0.7	-2.6, 1.2 [.461]
**Sleep efficiency**								
RSS	-0.04	-0.6, 0.6 [.908]	0.09	-0.7, 0.5 [.779]	0.2	-0.5, 0.8 [.597]	0.2	-0.4, 0.9 [.451]
PSS	-0.1	-0.5, 0.2 [.382]	-0.2	-0.5, 0.1 [.126]	0.02	-0.3, 0.3 [.919]	0.1	-0.2, 0.4 [.422]
PSAS	0.1	-0.2, 0.3 [.604]	0.03	-0.2, 0.3 [.824]	0.1	-0.1, 0.2 [.364]	0.1	-0.1, 0.2 [.424]
**Midpoint of sleep**								
RSS	-0.8	-5.9, 4.2 [.753]	-0.7	-6.0, 4.6 [.788]	-1.3	-4.6, 2.1 [.462]	-0.7	-4.0, 2.6 [.658]
PSS	-1.5	-4.0, 1.0 [.240]	-2.2	-4.7, 0.2 [.070]	-0.9	-2.6, 0.8 [.303]	-1.1	-2.7, 0.4 [.163]
PSAS	-2.8	-4.7, -0.9 [.004]	-1.2	-2.8, 0.3 [.124]	-0.6	-2.0, 0.9 [.464]	-0.5	-1.8, 0.9 [.500]

Estimated by linear mixed effects models. CI = confidence interval; RSS = relationship satisfaction scale; PSS = perceived stress scale; PSAS = pre-sleep arousal scale.

^a^ Adjusted for age, weekday and calendar month.

Exposure of total white light in the pregnant women, displayed in Table 4, was not associated with self-reported TST, SE or midpoint of sleep. For the actigraphy data, total white light was inversely associated with TST (B = -8.1, 95% CI = -14.7, -1.5) and earlier midpoint of sleep (B = -10.3, 95% CI = -14.7, -5.9). Total white light was not associated with SE.

**Table 4:** Association between sleep and evening light exposure in pregnant women.

	Self-reported				Actigraphy			
	Crude model:		Adjusted model:^a^		Crude model:		Adjusted model:^a^	
	Beta	95% CI [P]	Beta	95% CI [P]	Beta	95% CI [P]	Beta	95% CI [P]
**Total sleep time**								
Total white light	-2.0	-9.8, 5.8 [.608]	-2.4	-10.3, 5.5 [.558]	-11.4	-17.9, -4.9 [.001]	-8.1	-14.7, -1.5 [.016]
**Sleep efficiency**								
Total white light	0.7	-0.1, 1.6 [.088]	0.5	-0.4, 1.4 [.263]	0.2	-0.3, 0.8 [.364]	0.1	-0.4, 0.7 [.667]
**Midpoint of sleep**								
Total white light	-3.5	-9.6, 2.6 [.256]	-0.2	-5.2, 4.8 [.935]	-15.3	-20.0, -10.6 [< .001]	-10.3	-14.7, -5.9 [< .001]

Estimated by linear mixed effects models. CI = confidence interval; RSS = relationship satisfaction scale; PSS = perceived stress scale; PSAS = pre-sleep arousal scale.

^a^ Adjusted for age, weekday and calendar month.

## Discussion

The aim of this study was to assess sleep disturbance, sleep patterns and sleep related behavior in healthy pregnant women in the third trimester by comparing them to non-pregnant women. Further, the aim was also to investigate how evening light exposure and perceived stress were linked to the sleep of the pregnant women.

### Sleep in late pregnancy

According to sleep diary data the pregnant women had significant lower SE compared to the non-pregnant women in the adjusted analyses. According to the actigraphic data the pregnant women had higher TST and were exposed to more light the three last hours before bedtime compared to the non-pregnant group. None of the investigated stress variables were related to sleep outcomes, neither assessed by sleep diary or actigraphy among the pregnant women. Light exposure the three last hours before bedtime was inversely related to TST and midpoint of sleep according to the actigraphic data.

This study showed no difference between the two groups in terms of insomnia scores. The mean BIS scores of the two groups were actually quite comparable to national norm data for women in the relevant age groups [34]. Still, a relatively high proportion of the two groups fulfilled the criteria for insomnia, albeit no group differences emerged.

The non-pregnant group had a shorter total sleep time than the pregnant group measured by the actigraph, however this was not corroborated by self-reported data. This may reflect that the non-pregnant women overestimated sleep subjectively and/or that the pregnant women underestimated sleep. The time x group interaction for self-reported TST and midpoint of sleep suggested that the pregnant women slept relatively longer and later during the weekend compared to the non-pregnant women, hence they seemed to accumulate a greater sleep debt during the week compared to the non-pregnant women. Such a sleep pattern is common in working populations [43]. As the non-pregnant women comprised university students who probably have few early lectures and few mandatory tasks, the results may also reflect that this group to a large extent can sleep ad libitum many days per week [44]. The pregnant women reported significant lower SE than the non-pregnant women on the sleep diary, but not according to the actigraph. Sleep efficiency is often used as an indicator of sleep quality. Decreased sleep quality during pregnancy compared with a non-pregnant population has been reported previously [1]. The fact that this was not detected by actigraphy may suggest that movement during pregnancy is reduced. This is in line with studies suggesting that actigraphy may overestimate TST and SE in pregnancy when using default settings [45]. It should be noted that the mean age of the pregnant women was 30.6 years, compared to 23.7 years in the non-pregnant group.

The pregnant women were exposed to a higher level of total white light in the three last hours before bedtime compared to the non-pregnant women. This might reflect an earlier midpoint of sleep (although not significant difference between the groups in adjusted analysis), which can be an indicator of light exposure occurring earlier in the evening when natural illumination is at a higher level. The fact that the exposure of light was lower in the weekends, when the pregnant women went to bed later (results not shown), support this interpretation. In the pregnant women exposure to light according to actigraph data showed an inversely association with TST and midpoint of sleep, but not with SE. We did not find any association between light exposure and self-reported sleep variables.

The inverse relationship between light exposure before bedtime and TST suggests that high level of illumination may curtail sleep. This is in line with studies showing that light exposure in the evening may reduce sleepiness [16] and studies showing that light exposure disturbs sleep and circadian rhythms in mothers and their offspring [14].

In terms of stress, none of the stress scales (RSS, PSS and PSAS) showed any association with the sleep variables TST, SE and midpoint of sleep, both when assessed with sleep diary and actigraphy. Still, previous research has shown that psychosocial stress during pregnancy is associated with elevated risk of negative maternal health and birth outcomes [46] and women with stress-related sleep disturbances during pregnancy are more likely to experience insomnia [23]. Conversely, previous studies have shown that poor sleep quality can cause stress in pregnant women [7], both during second and third trimester of pregnancy [7, 47]. The present sample of pregnant women reported as a group, low scores on relationship-stress and other stress-related variables. A good relationship with partner contributes to low levels of perceived stress [32], which generally seems to protect against sleep problems [23, 24, 48]. In line with this, the present sample of healthy pregnant women in the first part of the third trimester slept overall quite well. Their overall sleep duration, both according to sleep diary and actigraphy was in line with the recommended amount for adults [49]. The mean SE was also above the suggested cut-off of 85%, commonly used to distinguish between sleep of acceptable quality and suboptimal sleep [50].

Overall, in the present sample of healthy pregnant participants we found that sleep was quite well preserved, in contrast to findings from several previous studies [1, 6]. However, the present sample was on average quite economical resourceful and reported low levels of exposure to stressors, which may explain the discrepancies with other studies. The data were collected in an early part of the third trimester, which in term of mechanical discomfort may be less challenging than closer to term.

### Limitations and strength

A limitation of the present study is the relatively small number of participants. Overall, the pregnant group reported good sleep and low exposure to stressors, either from their relationship with their partner and in general. Due to the low scores on sleep problems and stress the odds of finding a relationship between sleep and stress are assumingly quite low.

As chronotype might have moderated the findings, it is a limitation that subjects in the present study were not assessed by such measure. Mid-sleep time on weekend corrected for sleep debt on workdays is normally regarded as the best indirect subjective measure of circadian phase [51]. In the present study however, some had weekend work, some were on sick leave and some were students. We therefore used mid-sleep time averaged over the whole week as an indirect measure of circadian phase.

Only small and inconsistent differences were found between the sleep of the pregnant and the non-pregnant group, which shows that the pregnant group did not have poorer sleep than this non-pregnant group. Hence, the pregnant group seems healthier than other pregnant groups studied in terms of sleep and health. In this realm it is worth noticing that the pregnant group had higher education, higher income and had fewer minority members than in the general population. Thus, the generalizability of the findings may be limited. The comparative group comprised female students at the University of Bergen who were younger and likely have a different lifestyle than the pregnant women. This may pose a challenge when using these as a comparison group. Still, it should be noted that age was controlled for in the analyses. Also, a recent study [52], has shown a high prevalence of sleep problems among Norwegian students, hence the comparison group do probably not comprise good sleepers only.

Data on several clinical-demographic characteristics (marital status, education, economics, smoking and physical and relaxing activity) were unfortunately not collected in the student sample, precluding group comparisons on these variables. The comparative group did not complete the instruments PSAS, PSS and RSS. However, the scores on these instruments were not associated with sleep in the pregnant sample. The reliability in some instruments, like the PSS, was somewhat lower than ideally, with an alpha of .64. Some of the data in the present study were based on self-report which may render the data vulnerable to recall bias [53], social desirability bias [54], and common method bias [55]. Although actigraphy in many studies have shown low specificity [56], actigraphy has still shown to be sufficiently sensitive to detect changes in sleep duration in several studies [35]. A strength of the present study is the combination of subjective and objective sleep assessment, and an observation period of a full week. Despite that the light measure was not of sufficient quality to differentiate between different wavelengths and the fact that actigraph devices typically underestimates illuminance (both artificial and natural) [57], the inclusion of light measurement and analyses of associations with sleep parameters were of importance. Light was the only environmental factor showing association with sleep in the present study.

Another asset of the present study is that it contributes with knowledge suggesting that sleep, even at the end of pregnancy, may be fairly good in healthy and resourceful women. This is an important message to convey. In terms of future research there is a need for more knowledge about light exposure and its effects during pregnancy. Studies following sleep in pregnant women throughout the whole pregnancy would be an asset to the field. As sleep problems generally are common in pregnant women, more studies investigating the effects of non-pharmacological interventions are warranted.

## Conclusions

This study of healthy Norwegian pregnant women in the beginning of the third trimester showed that they as a group overall slept quite well. Compared to a non-pregnant group, there were few differences between the groups. In the present sample of pregnant women, light exposure in the evening was associated with shorter sleep duration. Perceived stress did not show any association with sleep parameters of pregnant women in the present study.

## Supporting information

S1 Dataset Table 1; demographic factors from selfreported data. (XLSX)

S2 Dataset Table 2; total sleep time, sleep efficiency and midpoint of sleep from selfreported data. (XLSX)

S3 Dataset Table 2; total sleep time from actiware data. (XLSX)

S4 Dataset Table 2; sleep efficiency from actiware data. (XLSX)

S5 Dataset Table 2; midpoint of sleep from actiware data. (XLSX)

S6 Dataset Table 2; total white light from actiware data. (XLSX)

S7 Dataset Table 3; total sleep time from selfreported and actiware data, and the stressors RSS, PSS and PSAS. (XLSX)

S8 Dataset Table 3; sleep efficiency from selfreported and actiware data, and the stressors RSS, PSS and PSAS. (XLSX)

S9 Dataset Table 3; midpoint of sleep from selfreported and actiware data, and the stressors RSS, PSS and PSAS. (XLSX)

S10 Dataset Table 4; total sleep time, sleep efficiency and midpoint of sleep from selfreported data, and total white light from actiware data. (XLSX)

S11 Dataset Table 4; total sleep time, sleep efficiency and midpoint of sleep, and total white light, all from actiware data. (XLSX)
